# Transcutaneous auricular vagus nerve stimulation to acutely reduce emotional vulnerability and improve emotional regulation in borderline personality disorder (tVNS-BPD): study protocol for a randomized, single-blind, sham-controlled trial

**DOI:** 10.1186/s13063-024-08230-6

**Published:** 2024-06-19

**Authors:** Giuseppe Guerriero, Sophie I. Liljedahl, Hanne K. Carlsen, Marta López Muñoz, Alexander R. Daros, Anthony C. Ruocco, Steinn Steingrimsson

**Affiliations:** 1 Section of Psychiatry and Neurochemistry, Institute of Neuroscience and Physiology, Sahlgrenska Academy, Gothenburg University, Gothenburg, Sweden; 2 National Specialized Medical Care Unit for Severe Self-Harm Behavior, Department of Psychiatry for Affective Disorders, Sahlgrenska University Hospital, Region Västra Götaland, Gothenburg, Sweden; 3 Centre of Registers, Region Västra Götaland, Gothenburg, Sweden; 4 Department of Psychiatry for Affective Disorders, Sahlgrenska University Hospital, Region Västra Götaland, Gothenburg, Sweden; 5 Department of Psychology, University of Windsor, Windsor, Canada; 6 Department of Psychological Clinical Science, University of Toronto, Toronto, Canada

**Keywords:** Transcutaneous vagus nerve stimulation, Borderline personality disorder, Emotional vulnerability, Emotion regulation

## Abstract

**Background:**

Borderline personality disorder (BPD) is considered a disorder of emotion regulation resulting from the expression of a biologically determined emotional vulnerability (that is, heightened sensitivity to emotion, increased emotional intensity/reactivity, and a slow return to emotional baseline) combined with exposure to invalidating environments. Vagal tone has been associated with activity in cortical regions involved in emotion regulation and a lower resting state of vagal tone has been observed in BPD patients relative to healthy controls. Non-invasive transcutaneous auricular vagus nerve stimulation (taVNS) has been shown to reduce temper outbursts in adults with Prader-Willi Syndrome, to enhance recognition of emotions in healthy students, and to improve depressive and anxiety symptoms. Furthermore, a single session of taVNS has been shown to acutely alter the recognition of facial expressions of negative valence in adolescents with MDD and increase emotion recognition in controls. However, the effect of taVNS on emotional vulnerability and regulation in individuals diagnosed with BPD has not been investigated. Our aims are to determine if taVNS is effective in acutely reducing emotional vulnerability and improve emotional regulation in BPD patients.

**Methods:**

Forty-two patients will be randomized to a single session of taVNS or sham-taVNS while going through an affect induction procedure. It will consist of the presentation of one neutral and three negative affect-evoking 4-min-long videos in sequence, each of which is followed by a 4-min post-induction period during which participants will rate the quality and intensity of their current self-reported emotions (post-induction ratings) and the perceived effectiveness in managing their emotions during the video presentation. The rating of the current self-reported emotions will be repeated after every post-induction period (recovery ratings). Mixed models with individuals as random effect will be used to investigate the ratings at each stage of the study, taking into account the repeated measures of the same individuals at baseline, pre-induction, post-induction, and recovery.

**Discussion:**

The study has potential to yield new insights into the role of vagal tone in emotion dysregulation in BPD and offer preliminary data on the effectiveness of taVNS as a possible non-invasive brain stimulation to treat a core symptom of BPD.

**Trial registration:**

ClinicalTrials.gov NCT05892900. Retrospectively registered on Jun 07, 2023.

## Introduction

### Background and rationale {6a}

Borderline personality disorder (BPD) is a severe and persistent mental disorder clinically characterized by affective instability and interpersonal, identity, cognitive, and behavioral disturbances [1]. Epidemiological studies indicate that BPD affects approximately 2–6% of the general population [2], with higher prevalence rates observed in clinical settings, where it accounts for an estimated 11% of outpatient cases and 19% of inpatient cases [3]. Albeit no sex differences have been reported in the prevalence of the disorder in the general population [2], 75% of BPD diagnoses in psychiatric outpatient clinics are among females [4].

According to Linehan’s Biosocial Theory of personality functioning [5], BPD is conceptualized as a condition characterized by difficulties in emotion regulation, stemming from a biologically rooted emotional vulnerability. This vulnerability encompasses heightened sensitivity to emotion, heightened emotional intensity and reactivity, and a slower return to emotional baseline. When coupled with an invalidating childhood environment, characterized by intolerance toward the expression of private emotional experiences, these factors contribute to the development of BPD [5].

The specific mechanisms underlying this emotional vulnerability remain unclear [6, 7]. Hypothesized biological causes may encompass a range of factors, including genetic influences, adverse intrauterine events, and early childhood environmental influences, like abuse and neglect [8], on the development of the brain and nervous system through epigenetic mechanisms [5, 9]. Studies using neuroimaging techniques have consistently shown alterations in the structure and function of regions such as the amygdala, prefrontal cortex, anterior cingulate cortex (ACC), and hippocampus in individuals with BPD [10]. Additionally, abnormalities in neurotransmitter systems, glutamatergic, serotonergic, dopaminergic, and noradrenergic, and in the opioid system have been implicated [11]. Furthermore, there is evidence suggesting alterations in the hypothalamic-pituitary-adrenal (HPA) axis, the primary stress response system in the body, in individuals with BPD [12].

At least two theories of emotions, the neurovisceral integration model (NIM) proposed by Thayer and Lane [13], and the polyvagal theory (PVT) by Porges [14], underscore the central role of the vagus nerve in regulating emotional processes and autonomic responses. The NIM posits a framework linking heart rate variability (HRV) to mental and physical health through the central autonomic network (CAN) [15]. This network integrates sensory, emotional, and cognitive information to regulate bodily functions. At the forebrain level, structures like the insular cortex, ACC, and amygdala integrate visceral sensations with motivational inputs. The hypothalamus further regulates homeostatic functions, while brainstem regions process pain and stress-related information. Within the CAN, sensory information is hierarchically processed before influencing motor output. Cortical areas, especially the prefrontal cortex, oversee regulatory processes. The NIM emphasizes cortical control in emotional regulation, linking deficits in cortical function to inflexible emotional responses[13]. The CAN’s primary autonomic output affects heart function, with HRV serving as a valuable index of autonomic balance. Greater parasympathetic activity, reflected in higher HRV, is linked to increased activations in the prefrontal cortex and ACC and indicates effective cortical-inhibitory control and self-regulation [16–18].

The PVT offers an evolutionary perspective on the development of the autonomic nervous system (ANS). According to this theory, the ventral myelinated aspect of the vagus nerve, which evolved more recently, evolved to support social affiliations and interactions [19]. In safe environments, the vagus nerve suppresses primitive defense reflexes of the sympathetic system, favoring more efficient and soothing functions, known as the “social-engagement system” [20]. This activation leads to enhanced situational judgment, flexible behaviors, and pro-social traits, facilitating the formation of organized societies and communal structures [14]. Additionally, the PVT introduces the concept of neuroception, where the vagus nerve integrates interoceptive, somatosensory, and endocrine information to subconsciously determine environmental danger [19]. Altered interoceptive pathways can result in abnormal neuroceptive states, inflexible emotional responses, and irregular vagal outputs [13]. The ventral area of the vagus nerve acts as a regulatory “brake,” maintaining balance at rest and mobilizing energy through the sympathetic nervous system when needed [21].

Furthermore, studies have demonstrated that lowered resting state vagal tone, as indicated by reduced vagal-mediated heart rate variability (vmHRV), is consistently linked to difficulties in emotion regulation and heightened impulsivity [18, 22, 23]. This association has been investigated using various methodologies, including psychophysiological measures such as HRV and electrodermal activity, as well as self-report measures assessing emotion regulation strategies and impulsive tendencies [18, 22, 23]. A lower vmHRV has been consistently observed in individuals with a range of psychiatric disorders, including BPD, compared to healthy controls [24, 25]. This finding has led researchers to propose a transdiagnostic psychophysiological mechanism underlying the relationship between vagal tone and difficulties in emotion regulation and impulsivity. Specifically, it is suggested that alterations in vagal tone may disrupt the balance of autonomic nervous system activity, leading to dysregulated emotional responses and impulsive behaviors [26]. Moreover, it has been suggested that BPD patients engage in self-harm to increase vagal tone in prefrontal areas resulting in improved regulation of emotions [27].

Collectively, these findings suggest that electrical stimulation of the vagus nerve (VNS) holds promise for reducing emotional vulnerability and enhancing emotion regulation in individuals with BPD. This potential effect may arise through two primary mechanisms: First, VNS may augment vagal tone and activate cortical regions involved in top-down emotion regulation. Second, it may enhance the processing of afferent interoceptive signals transmitted via the vagus nerve to key brain regions including the brainstem, limbic system, and cortex. Notably, a proposed frontal-vagal network, which encompasses functional nodes such as the dorsolateral prefrontal cortex (dlPFC), subgenual anterior cingulate cortex (sgACC), and the vagus nerve, has emerged as a target for neuromodulation therapies in depression. This network’s ability to modulate heart rate and heart rate variability (HRV) underscores its potential significance in regulating emotional states [28]. Additionally, research indicates that vagus nerve stimulation can induce rapid effects on sensory processing, distinct from the long-lasting effects observed with repeated stimulation, as evidenced in both animal and human studies [29].

Surgically implanted vagus nerve stimulation (iVNS) was approved in 2001 as an adjunct long-term treatment for treatment-resistant depression by the European Medicines Agency (EMA), and in 2005 by the Food and Drug Administration (FDA). Electrical stimulation of the vagus nerve provides stimulation to the nucleus tractus solitarii (NTS), which in turn modulates multiple regions of the brain via its neuronal connections to anatomically distributed subcortical and cortical regions of the brain. However, iVNS requires the surgical implantation of a pulse generator underneath the skin, connected to an electrode placed onto one of the branches of the cervical part of the vagus nerve [30]. Surgical risks, technical challenges, and potential side effects have limited the use of iVNS in clinical practice.

Non-invasive transcutaneous vagus nerve stimulation (tVNS) methods have been developed to mitigate the risks of iVNS. There are currently two major ways to use tVNS. The first is to apply stimulation by using two skin electrodes by a hand-held device (e.g., gammaCoreTM, electroCore, Inc.) to the cervical portion of the vagus nerve (transcutaneous cervical vagus nerve stimulation or tcVNS) and the second is to apply stimulation by using two surface electrodes (e.g., tVNS^®^, tVNS Technologies GmbH) to the ear (transcutaneous auricular vagus nerve stimulation or taVNS). In addition to these uses, a minimally invasive form of percutaneous auricular VNS (paVNS) can be performed with 2–3 miniature needle electrodes penetrating the skin in the targeted outer ear regions innervated mainly by the auricular branch of the vagus nerve (e.g., Auristim, DyAnsys) [31]. The reason for ear stimulation (taVNS) is focused on anatomical studies that show afferent vagus nerve distribution in some parts of the ear (concha and lower half of the back ear over the mastoid process). It is thought that stimulation of the vagus nerve’s auricular branch will stimulate the inferior ganglion, which projects to the NTS, and thus produce similar therapeutic effects to iVNS [32]. Several functional magnetic resonance imaging (fMRI) studies investigating the effects of taVNS on brain activity in healthy individuals have supported this hypothesis, revealing consistent activation of NTS and locus coeruleus [33–37]. Moreover, taVNS induced changes in limbic structures such as the amygdala, hippocampus, prefrontal cortex, and ACC, which are part of depression-related neural circuits and crucial for emotion processing and regulation [34–36]. Specifically, increased activation was observed in regions such as the insula, precentral gyrus, thalamus, prefrontal cortex, and ACC while decreased activation was noted in the amygdala, hippocampus, and parahippocampal gyrus following taVNS [35, 36].

In major depressive disorder (MDD), one of the major applications of iVNS, taVNS has been shown promising results affecting brain connectivity and neurotransmitter concentrations. Li et al. [38] observed increased connectivity between the left rostral anterior cingulate cortex (rACC) and several brain regions, alongside reduced GABA and glutamate levels in treatment-resistant MDD patients receiving taVNS and sertraline. Tu et al. [39] reported decreased connectivity between the bilateral medial hypothalamus and rACC in the taVNS group, correlating with symptom improvement. Wang et al. [40] found increased connectivity between the left nucleus accumbens and bilateral medial prefrontal cortex (mPFC)/rACC, associated with symptom alleviation. Additionally, altered connectivity within the default mode network and salience network was observed in the taVNS group, along with enhanced connectivity between the right amygdala and left dlPFC, linked to symptom reduction and anxiety improvement [38–40]. These results are paralleled by several studies showing a significant effect of taVNS on depressive and anxiety symptoms [41–46]. A recent meta-analysis including 12 randomized controlled trials (RCTs) with totally 838 subjects evaluated the efficacy and safety of taVNS in depression treatment [46]. The results showed that patients treated with taVNS exhibited significantly higher response rates and larger reductions in depression scores of large effect-size compared to those receiving sham taVNS. Notably, taVNS demonstrated a significantly higher response rate than antidepressants, albeit with a small effect size on depression scores. Additionally, while response rates were similar, the taVNS combined with the antidepressants group showed larger reductions in depression scores compared to the antidepressants-only group. However, the quality of evidence was low to very low [46].

The efficacy of taVNS has been investigated also in epilepsy, since iVNS has become an important adjunctive therapy for refractory cases [47]. A meta-analysis, encompassing three RCTs with a total of 280 patients, revealed a significant difference in seizure frequency between the treatment and control groups. The treatment group exhibited a mean overall reduction of 42% [48], which was similar to the 44.6% achieved with iVNS [49]. However, due to insufficient study data, the authors were unable to draw a conclusion regarding a significant difference in responder rates [48]. In addition to depression and epilepsy tVNS is also being studied for a variety of illnesses, including headache, tinnitus, atrial fibrillation, schizophrenia, and chronic pain [32].

The mentioned tVNS approaches involve various devices and stimulation protocols [32]. There is currently no strong data regarding the location and type of stimulation that is required to achieve a therapeutic effect. The most often used devices are gammaCore^TM^ for stimulation at a neck site (FDA-authorized for acute and/or prophylactic treatment of primary headache) and tVNS^®^ for stimulation of the auricular branch of the vagus nerve on the ear (CE-marked for its use in epilepsy, depression, anxiety, pain, and migraine). Most devices are battery-powered control units that allow patients to administer tVNS at home while doing other activities (for example, listening to music). The stimulation parameters used in studies of depressed patients range in stimulation frequency from 1.5 to 120 Hz, while the duration of stimulation ranges from 15-min at a frequency of 5 times per week, to 30-min at a frequency of twice per day [32]. Furthermore, the ability of taVNS to modulate vmHRV remains unclear due to inconsistent results observed among studies [50–54]. A meta-analysis conducted using a Bayesian random-effects model on 16 single-blind studies comparing taVNS with sham taVNS in healthy participants showed compelling evidence for the absence of an effect of taVNS on vmHRV [54]. Explanations for these results, besides a genuine lack of effect, include the direct stimulation of afferent projections of the vagus nerve to the NTS, which only indirectly modulate heart rate, differences in stimulation amplitude, potentially attenuating group-level effects compared to sham stimulation, and the absence of studies administering taVNS on the right side [54].

In animal studies, the stimulation of the vagus nerve through iVNS has been shown to produce an immediate dose-dependent anxiolytic effect [55] and to enhance fear extinction and reduce anxiety in rat models of PTSD, after both, moderate [56, 57] and prolonged and repeated trauma [58]. In humans, taVNS has been shown to reduce anger outbursts in adults with Prader-Willi Syndrome [59], enhance recognition of emotions in healthy students [60], and improve depressive and anxiety symptoms in adults and adolescents with clinically-diagnosed depression albeit with uncertain evidence due to the scarcity of available controlled studies [41]. Moreover, 4 weeks of taVNS has been shown to increase amygdala functional connectivity in patients with depression in comparison with sham taVNS [61]. A single session of taVNS has been shown to acutely alter the recognition of briefly presented facial expressions of negative valence in adolescents with MDD and increase emotion recognition in controls [62], as well as boost mood after prolonged effort exertion in healthy participants [63], in comparison with sham taVNS. In sum, taVNS has been shown to be safe and well-tolerated in humans at the doses tested, with local skin irritation being the most common side effect, followed by headache and nasopharyngitis [64].

To our knowledge, the effect of taVNS on emotional vulnerability in individuals diagnosed with BPD has never been investigated.

### Objectives {7}

The primary objective of the study is to assess the efficacy of one taVNS session compared to sham control to acutely reduce emotional reactivity in BPD patients. The secondary objectives are to assess the efficacy of one taVNS session compared to sham control to acutely (a) reduce baseline emotional arousal, (b) ease emotional recovery, and (c) improve emotional regulation in BPD patients.

### Trial design {8}

This study will be conducted in accordance with SPIRIT guidelines for clinical trial protocols. The study will be a randomized, single-blind, sham-controlled trial with two parallel groups and repeated measures design.

Participants will be randomized to undergo a single 45-min session of either taVNS (intervention group) or sham-taVNS (control group) while undergoing an affect induction procedure. It will consist of the presentation of four (one neutral and three negative-affect-evoking) 4-min-long videos in sequence, each of which is followed by a 4-min post-induction period. The participants will rate the quality and intensity of their current self-reported emotions at baseline, after 4-min taVNS (pre-induction ratings), after each video (post-induction ratings), and after 4 min after each video (recovery ratings) (Table 1).

**Table 1:**
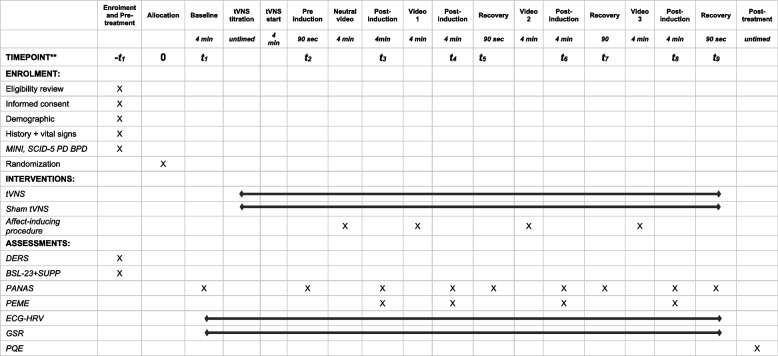
Schedule of enrolment, interventions, and assessments

## Methods: participants, interventions, and outcomes

### Study setting {9}

The study will be conducted on 42 outpatients with an established borderline personality disorder (BPD) diagnosis according to Diagnostic and Statistical Manual of Mental Disorders-Fifth Edition (DSM-5). The participants will be recruited among those having an ongoing contact or discharged from a department of psychiatry in Sweden.

### Eligibility criteria {10}

Participants will be included in this study if they meet the following criteria: Swedish-speaking and able to provide informed consent to participate in the study; Female and between the ages 18 and 50 years old (the decision to include only women in the study is motivated by the higher frequency of BPD diagnosis in women and by the need to have a homogeneous study sample. The inclusion of men in future studies is not excluded); Current DSM-5 diagnosis of BPD based on the Structured Clinical Interview for DSM-5 Personality Disorders (SCID-5-PD); Capable (in the Investigator’s opinion) and willing to comply with all study requirements.

The participant will be excluded from the study if any of the following apply: any unstable medical and/or neurological condition; currently pregnant; any significant neurological disorder or condition likely to be associated with increased intracranial pressure or cognitive impairment (e.g., a space occupying brain lesion, a history of stroke, a cerebral aneurysm, a seizure disorder, Parkinson’s disease, Huntington’s chorea, multiple sclerosis); current diagnosis of delirium, dementia or another cognitive disorder secondary to a general medical condition; established diagnosis of a developmental and neuropsychiatric disorder (e.g., Down syndrome, autism-spectrum disorder, ADHD); non-correctable clinically significant sensory impairment (i.e., cannot hear or see well enough to complete the affect induction procedure, follow and answer the survey instructions and questions); alcohol or substance use disorder (relating to opioids, cocaine, amphetamine or benzodiazepine) currently or within the past 1 month; daily treatment with antiepileptics (e.g., carbamazepine, gabapentin, lamotrigine, levetiracetam, pregabalin, sodium valproate, topiramate) or benzodiazepines (last dose within 7 days before the screening to prevent eventual dampening of the central nervous system and/or vagus nerve excitability); intracranial implant (e.g., aneurysm clips, shunts, stimulators, cochlear implants, or electrodes) or any other metal object within or near the head, excluding the mouth, that cannot be safely removed; history or diagnosis of bipolar or chronic psychotic disorder (e.g., schizophrenia, schizoaffective disorder).

The study will be performed by medical doctors.

### Who will take informed consent? {26a}

Potential candidates will be referred to the study team by their healthcare providers or recruited on a voluntary basis by on-site advertisements. The interested candidates will be contacted by the physician (investigator) over the phone to be informed about the study nature, to evaluate the potential interest in participating in the study, and to preliminarily evaluate their eligibility. A copy of the informed consent will be sent via e-mail, by ordinary mail, or given on-site to the potential participants to give them time to read it before being invited to the laboratory and make a final decision about the eventual participation in the study. If interested, the participants will be invited to the lab to have further clarifications about the study procedure and their participation to the study and eventually sign the informed consent. The physician (investigator) will obtain informed consent from potential trial participants. The participant must personally sign and date the latest approved version of the informed consent form before any study-specific procedures are performed.

### Additional consent provisions for collection and use of participant data and biological specimens {26b}

N/A. No other consents are needed from participants since the data collected will be used for the sole purpose of the study and no biological specimens will be collected.

## Interventions

### Explanation for the choice of comparators {6b}

**Fig. 1:**
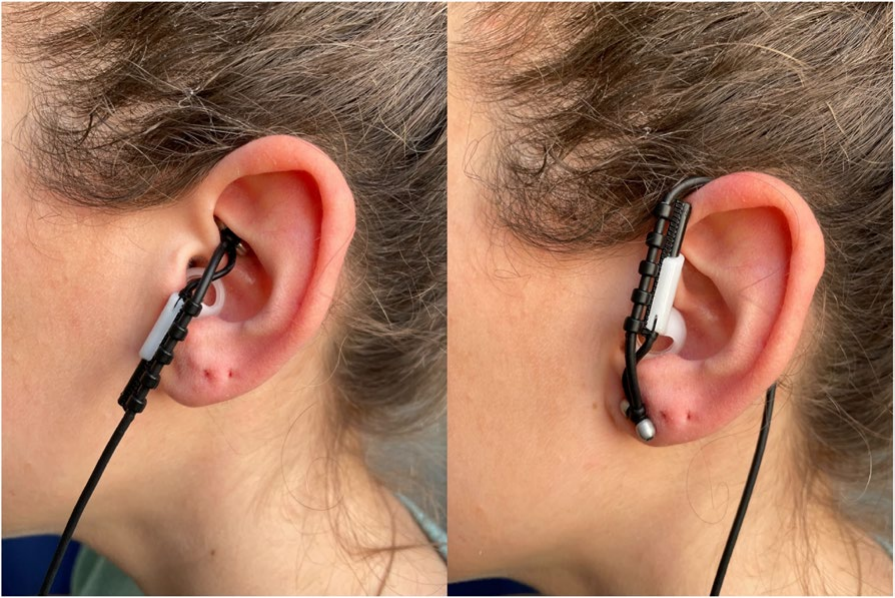
Positioning of the stimulation electrodes in the active (left) and in the sham (right) condition

Participants will be randomized to the active taVNS or sham taVNS. To reach an effective stimulation of the vagus nerve through taVNS in the active condition, the electrodes will be placed at the left ear concha. The ear concha is principally innerved by the afferent branch of the vagus nerve (ABVN) [52]. In the sham taVNS condition, the stimulation electrodes will be placed at the center of the left ear lobe, which is known to be free of cutaneous vagal innervation [65] and the electrical stimulation at this anatomical area is not able to activate the vagus pathway [66] (Fig. 1). This method was chosen because it has the advantages that it has already been used in several sham-controlled studies [60, 67–69], it is easy to use, and the participants can still feel a tickling sensation as for the active taVNS, making it more difficult to distinguish between the active and the sham conditions. The disadvantages are that the participants may have seen pictures of the active taVNS electrode placing leading to a possible unblinding and that the innervation of the ear lobe is still not completely clear leading to possible unwanted effects.

### Intervention description {11a}

The participants will sit comfortably in front of a computer monitor, with an adjustable height chair, and will be reminded of the potential provocative nature of the task (i.e., that the clips will contain profane language, sexual content, and violence). Three electrocardiographic (ECG) electrodes will be applied at the right collar and at the right and left lowest rib cage bones and two galvanic skin response (GSR) electrodes will be placed on the volar proximal phalanges of the fingers on the palms of the non-dominant hand. Participants will be instructed to maintain their visual attention to the computer monitor, avoid closing their eyes as much as possible, and will be told that the task is simply to immerse themselves into each video. Participants will be provided over-the-ear headphones, and the lights will be dimmed to further immerse participants into the videos. An investigator/research assistant will always remain seated quietly beside the participant but out of the participant’s direct view.

A continuous ECG and GSR recording for the entire procedure will start and a baseline assessment of the current self-reported emotions using the Positive and Negative Affect Schedule (PANAS) [70], will be collected.

After the baseline assessment has been completed, the transcutaneous electrical vagus nerve stimulation, delivered through a taVNS device (tVNS® R - tVNS technologies, Germany), will start. First, the taVNS device will be described and the taVNS electrode positioned at either the ear concha (active taVNS) or ear lobe (sham taVNS). The device consists of a stimulation unit and an ear electrode that is worn like an earphone. The stimulation unit sends electrical impulses through the electrode, which stimulates a branch of the vagus nerve transcutaneously (through the skin) in the auricle. The device can stimulate at an intensity between a minimum of 0.1 mA to a maximum of 5.0 mA with a biphasic waveform, an impulse duration of 28 s on and 32 s off, and an impulse frequency of 25 Hz. The stimulation intensity will be adjusted for every subject gradually by 0.1 mA at time until a tingling sensation or pulsation can be felt at the stimulation location. The stimulation should be clearly noticeable but not painful or uncomfortable. The device is CE-marked, indicating conformity with European Union regulations for medical devices, for the following conditions: anxiety, asthma, atrial fibrillation, autism, cognitive impairment, Crohn’s disease, depression, epilepsy, fibromyalgia, inflammation, migraines, Parkinson’s, Prader-Willi yndrome, sleep disorders, stroke, tinnitus. However, it is important to clarify that while the device has obtained CE marking, it has not received approval from the FDA or the EMA for the treatment of the listed conditions.

Four minutes after the optimal stimulation dose has been reached, the affect induction procedure will start. The affect induction procedure that will be used in this trial was developed by Daros and colleagues [71]. It has been shown to be tolerable and able to evoke higher negative emotional arousal in BPD patients in comparison with healthy controls [71]. It consists in the presentation on a computer screen of four (one neutral and three affect evoking) 4-min-long videos in sequence, each of which is followed by a 4-min post-induction period during which participants will rate the quality and intensity of the emotions perceived (PANAS) (post-induction ratings) and the perceived effectiveness in managing their emotions (PEME) [32].

The neutral video depicts a mother attempting to show a father that their young son can play chess and elicited very low levels of emotions in an undergraduate sample [72]. The three negative clips depicted a scene of domestic abuse between a man and a pregnant female partner; a funeral scene where a young girl struggles with the death of her friend; and a scene involving sexual assault by a police officer toward a woman while her husband watches. The domestic abuse clip significantly increased negative mood in a community-sourced sample [73]. The funeral clip elicited sadness in an undergraduate sample, whereas the sexual assault clip elicited anger, disgust, and contempt [72].

The titles and years of production of the films from which the videos are taken are *Searching for Bobby Fischer* (1993; neutral), *Nil by Mouth* (1997; domestic violence), *My Girl* (1991; funeral), and *Crash* (2004; sexual abuse).

After every post-induction period, for the three emotion-inducing videos, the participants will have other 90 seconds to rate again the PANAS (recovery ratings) (see Table 1). The neutral video will be always presented first to allow participants to orient themselves to the procedure. The three affect-evoking videos will be instead presented in a random order using the “randomizer” function of a Qualtrics software [74] (see below). Each page will be locked with a visible timer to ensure that all participants will progress through the procedure at the same pace. If a participant finishes the questions in advance, he/she will be instructed to sit quietly until the next page appears.

At the end of the survey, participants will answer additional questions on the quality of their participation (PQE).

All the instructions, questions, videos, and rating scales will be administered through an online Qualtrics survey [74], with time-fixed auto-advance according to the protocol-defined exposition time for every step of the experiment (see Table 1).

The software IMotions [75] will be used to integrate the data collected from the Qualtrics Survey and from the ECG and GSR sensors.

### Criteria for discontinuing or modifying allocated interventions {11b}

Each participant has the right to withdraw from the study at any time. In addition, the investigator may discontinue a participant from the study at any time if the investigator considers it necessary for any reason, including ineligibility (either arising during the study or retrospective having been overlooked at screening), significant protocol deviation, significant non-compliance with study requirements, an adverse event which requires discontinuation of the taVNS or results in inability to continue to comply with study procedures, emotional crisis which results in inability to continue to comply with study procedures or consent withdrawn.

The reason for withdrawal will be recorded in the electronic Case Report form (eCRF) (Qualtrics module) and in a research note in the patient research diary. If the participant is withdrawn due to an adverse event, the investigator will arrange for follow-up visits or telephone calls until the adverse event has resolved or stabilized.

### Strategies to improve adherence to interventions {11c}

The investigator will sit in the experiment room together with the participant monitoring all the phases of the experiment and be available to help with any eventual problems or difficulties that may emerge in complying with the study procedure. All the instructions and the outcome scales will be administered on a computer screen using a Qualtrics survey with fixed time, enough to read the instructions and answer all the questions, and auto-advance (participants will not be able to advance to the next question by themselves). The participant will sit comfortably in front of a computer screen wearing a headset to reduce the influence of potentially distracting environmental noise and facilitate the immersion in the task. A continuous ECG will be recorded throughout the experiment and an HRV analysis will be performed to check if taVNS was able to activate the vagus nerve in the active group. The galvanic skin response (GSR) will be also measured throughout the entire procedure to control that the affect-inducing procedure was able to generate emotional arousal independently of the participants’ self-scores for emotional arousal. After the experiment completion, participants will be asked to evaluate the quality of their participation with questions on the perceived empathy with the video characters, eventual avoidance behaviors during the affect evoking procedure, the impact of the presence of the investigator in the room on their ability to attend the procedure, the level of attention during the task, the presence of eventual distractors or other barriers, suggestions to reduce the eventual burden of participation.

### Relevant concomitant care permitted or prohibited during the trial {11d}

Participants consuming medications other than antiepileptics and benzodiazepines will be allowed to continue to consume them. No change in medications is required to participate in the study. Any medication taken during the study will be recorded in the eCRF (anamnestic module of the Qualtrics survey). Participants with an ongoing treatment with antiepileptics and benzodiazepines will be excluded from the study.

### Provisions for post-trial care {30}

If the participant is withdrawn due to an adverse event, the investigator will arrange for follow-up visits or telephone calls until the adverse event has resolved or stabilized. Eventual harm from trial participation will be covered by the Swedish ordinary patient injury insurance.

### Outcomes {12}

The primary outcome will be the change in negative emotional arousal from baseline, assessed immediately after the affect-induction procedure (post-induction ratings) using the PANAS. Negative emotional arousal will be measured through self-reported ratings of negative emotions on the PANAS (PANAS-N) at five time-points: baseline, after the neutral video, and after each of the three affect-inducing videos (designated as t1, t3, t4, t6, and t8 in Table 1). The means of the PANAS-N scores for each time point will be calculated and used to test the null hypothesis of no difference between the taVNS and sham taVNS groups. A mixed model analysis will be employed to assess each time point separately, ensuring comprehensive evaluation across all time points. The PANAS-N scores have already been used to measure the emotional arousal in previous studies using the same affect induction procedure with BPD patients and have been shown to be able to detect within and between groups differences [28]. The GSR recording, a physiological measure of emotional arousal, will be performed throughout the entire procedure and used to validate the PANAS-N data.

Since an increased emotional reactivity/intensity, one of the three components of the emotional vulnerability together with emotional sensitivity and longer emotional recovery time, is central in the physiopathology of BPD and the underpinning for many of the related symptoms and dysfunctional behaviors (e.g., self-harm) [5], to test the effect of taVNS on this primary outcome may have direct clinical utility. The available psychological treatments are associated with an improvement in the emotional regulation of BPD patients but have uncertain effects on the underlying emotional vulnerability [33].

The secondary outcomes of the study include:

- Change in negative emotional arousal from baseline at prior to affect induction (pre-induction ratings) as assessed by PANAS.
- Change in negative emotional arousal from immediately after affect-induction at 4 min after affect induction (recovery ratings) as assessed by PANAS
- Perceived effectiveness in managing emotions (PEME) scores (0–9) during affect induction.

The change in negative emotional arousal from baseline at prior to affect induction measured through the PANAS-N scores has been included as a secondary outcome to test if taVNS can acutely reduce the baseline emotional arousal. It has been reported that BPD patients have higher resting-state emotional arousal levels, possibly explaining the elevated sensitivity to perceive emotion activation (i.e., emotion sensitivity) even with small emotional triggers [6]. The means of the PANAS-N scores taken at baseline (t1) and 4 min after the taVNS/sham taVNS has begun, before the affect induction procedure (t2) (pre-induction scores), will be used to test the null hypothesis of no difference between the taVNS and sham taVNS groups.

In the same way, the change in negative emotional arousal from immediately after affect-induction at 4 min after affect induction has been chosen to test the effect of taVNS on the third component of emotional vulnerability (i.e., slow return to emotional baseline). The emotional arousal will be measured through the PANAS-N at six time-points, i.e., immediately after (t4, t6, and t8) and at 4 min after each of the three affect-inducing videos (t5, t7, and t9). The means for the PANAS-N scores for each time point in which they are assessed will be used to test the null hypothesis of no difference between the taVNS and sham taVNS groups.

The last secondary outcome will be the PEME during affect induction. Participants will be asked during the postinduction period for each of the four videos (t3, t4, t6, and t8) about their perceived effectiveness in managing their emotions by asking them to rate “How difficult was it to manage your emotional response to this film clip?” from 1 = not at all to 9 = extremely. This scale will be interpreted as a subjective difficulty in regulating emotions in response to each video stimulus as done by Daros and coworkers [28].

### Participant timeline {13}

The participant timeline is shown in Table 1.

### Sample size {14}

We are going to recruit 42 participants (21 participants per arm). The total sample size has been calculated using G*Power 3.1 Software to test an effect size of 0.25 with a power (1−*β*) = 0.8 for a sample with two independent groups (taVNS vs Sham-taVNS) and 4 dependent measures (PANAS-N score post-induction for neutral, funeral, sex-assault and domestic violence videos). The sample size needed is *n* = 34, calculated with an alpha level of 0.05. With an expected level of attrition of about 20%, the sample size has been increased by a factor of 1/(1–0.2) = 1.25 or 25% to 42 subjects.

## Recruitment {15}

The participants will be recruited among those having an ongoing contact or discharged from a psychiatry department in Sweden. The presence of a dedicated outpatient unit for personality disorders within the department of psychiatry for affective disorders at Sahlgrenska University Hospital in Gothenburg, with circa 1000 ongoing patients, is expected to speed up the recruitment process to reach the target sample size. Potential candidates will be referred to the study team by their healthcare providers or recruited on a voluntary basis by on-site advertisements. The interested candidates will be contacted by the investigator over the phone to be informed about the study nature, to evaluate the potential interest in participating in the study, and to preliminarily evaluate their eligibility. A copy of the informed consent will be sent via e-mail, by ordinary mail, or given on-site to the participants to give them time to read it before being invited to the laboratory and make a final decision about their eventual participation in the study.

## Assignment of interventions: allocation

### Sequence generation {16a}

The computerized random number generator is used to generate random sequences on a 1:1 basis. The random sequence will be put into a sealed, opaque, and sequentially numbered envelope by a team member not involved in the enrolment and investigation procedures. When the participant is admitted to the operating room the investigator will sequentially open the envelopes, based on their numbered order, to obtain a random sequence to determine the grouping. Participants with random sequences 1–21 are assigned to group taVNS, while 22–42 are assigned to group sham taVNS. Each participant will be given a unique study sequential number on the envelope cover in the order of their participation in this study. The random sequences of all participants and their corresponding study sequential numbers will be recorded.

### Concealment mechanism {16b}

The use of opaque sealed envelopes will ensure concealment until the interventions are assigned.

### Implementation {16c}

A member of the research team not involved in the enrolment and investigation processes will generate the allocation sequence using a computer program and seal it in opaque envelopes. The envelopes will be handed over to the investigator who will open them according to the unique sequential number on the envelope at the intervention allocation.

## Assignment of interventions: blinding

### Who will be blinded {17a}

Only the trial participants will be blinded (single-blind). The two treatments taVNS and sham taVNS will be potentially indistinguishable for participants but not for investigators, since both groups will perceive the electrical stimulation even if at two different locations at the ear (concha for taVNS and ear lobe for sham taVNS). The eventual collection of information by the participant on the taVNS method and the correct positioning of the electrode to reach a stimulation of the vagus nerve could lead to a potential unblinding for the participants too.

## Procedure for unblinding if needed {17b}

In the event of an emergency, the investigator is to decide the necessity of unblinding the subject’s treatment assignment. If unblinding occurs, the investigator must record the reason for unblinding, as well as the date and time of the unblinding.

## Data collection and management

### Plans for assessment and collection of outcomes {18a}

#### Data collection

A Qualtrics Survey [31] module will be the electronic case report form (eCRF) and used as the source document for the anamnestic information collected at the screening and for all the baseline and subsequent assessments and evaluations with the exceptions of the two diagnostic interviews MINI and SCID-5-PD, that will be administered by the investigator in paper format.

#### Screening

The screening procedure will be completed at the lab after the informed consent is signed and before the study procedure will start, to check the eligibility to the study and collect background information that will be used to analyze and interpret the study results. The following information will be collected and recorded: Demographics (date of birth, gender), Medical History (details of any history of disease or surgical interventions), Concomitant Medication (all over-the-counter or prescription medication, vitamins, and/or herbal supplements); Physical Examination (height, weight, and oral temperature; resting pulse and blood pressure (BP) measurements will be measured after the participant has sat for at least 5 min). Anamnesis of the substances assumed before the procedure (drugs, alcohol, nicotine, caffeine). No blood or urine samples will be collected.

The BPD module of the SCID-5 PD and MINI will be administered to confirm the BPD diagnosis and exclude the diagnosis of bipolar disorder, alcohol or substance use disorder, and history or diagnosis of bipolar or chronic psychotic disorder (e.g., schizophrenia, schizoaffective disorder).

#### Borderline personality disorder general psychopathology

The participants will complete the following additional self-report measures to assess BPD symptoms, self-harm, and emotion dysregulation severity:

- Difficulties in Emotion Regulation Scale (DERS) to assess difficulties in emotion regulation. The DERS [34] is a 36-item self-report measure of six facets of emotion regulation. Items are rated on a scale of 1 (“almost never [0–10%]”) to 5 (“almost always [91–100%]”). Higher scores indicate more difficulty in emotion regulation [34].
- Borderline Symptom List (BSL-23) and Borderline symptom list - behavior supplement (BSL-SUPP) to quantify symptoms and behaviors associated with BPD. The Borderline Symptom List – Short Version (BSL-23) is a 23-item self-rating instrument for specific assessment of borderline personality disorder (BPD) symptomatology in adults (18+). The scale assesses DSM BPD diagnostic criteria (e.g., affective instability, recurrent suicidal behavior, gestures, or threats, or self-mutilating behavior, and transient dissociative symptoms) in addition to items that are based on borderline-typical empirical findings regarding self-criticism, problems with trust, emotional vulnerability, and proneness to shame, self-disgust, loneliness, and helplessness [35]. The BSL-23 has a single factor structure and excellent psychometric properties, with high internal consistency with a Cronbach’s of 0.97 and test–retest reliability of 0.82 within 1 week [36]

#### Self-reported rating measures

The primary outcome will be measured through the PANAS [27]. Before undergoing the taVNS and affect induction procedure, (“baseline” or t1), after 4-min taVNS and before the first video (“preinduction” or t2), and after each video (“postinduction” or t3, t4, t6, and t8), participants will be asked to provide affect ratings using the 20-item PANAS [27] (Table 1). The scale uses adjectives that describe mood states rather than discrete emotions and are rated from 1 = very slightly or not at all to 5 = extremely. The PANAS is used widely in mood induction procedures because of its good validity and test–retest reliability. At postinduction, participants provided self-report ratings on six basic emotions felt in response to each video (disgust, fear, anger, sadness, happiness, and surprise) on a scale from 1 = not at all to 7 = extremely. A total of 4 min after each negative video, participants provided another set of PANAS ratings to measure changes in mood over a longer period (“recovery” or t5, t7, and t9). The PANAS is a reliable and valid measure of the construct it is intended to assess [37].

Moreover, participants will be asked during postinduction about their perceived effectiveness in managing their emotions (PEME) by asking them to rate “How difficult was it to manage your emotional response to this film clip?” from 1 = not at all to 9 = extremely. This scale will be interpreted as a subjective difficulty in regulating emotions in response to each video stimulus.

#### Physiological measurements

During the entire procedure, continuous ECG recording in a sitting position and a GSR will be performed using IMotions software [32] to integrate the sensors’ data with the affect-induction procedure and the self-reported measures.

For the ECG recording, a 3-electrodes Shimmer 3 ECG device will be used. The electrodes will be placed at the right arm, right leg, and left leg. The ECG recording will be used to measure the HRV variability as a potential indicator of actual stimulation of the vagus nerve in the taVNS group and as an additional and indirect measure of emotion regulation in both groups.

For the recording of electrodermal activity (EDA, also known as galvanic skin response; GSR), recording will use a 2 electrodes Shimmer GSR device. The GSR electrodes will be placed on the volar phalanges of the fingers (proximal, medial, or distal) on the palms of the non-dominant hand. The GSR will be used to give an additional objective physiological measure of emotional arousal/intensity that in our hypothesis will parallel the PANAS ratings given by the participants.

#### Participation Quality Evaluation

After the completion of the stimulation procedure and assessment, the following additional questions will be administered to participant to evaluate the quality of their participation (Participation Quality Evaluation or PQE): *To what extent did you empathize with the characters in the film? (Repeated for all the four videos); Did you close your eyes during any of the videos in this part of the experiment? Did you feel extremely uncomfortable with regards to the content in the videos? Did you feel that you let yourself experience the emotions that you were experiencing? Was it difficult to do this because someone else was in the room with you? Had you seen any of the video clips used in this study before? How was your attention during the procedure? Was there anything distracting? Were there other barriers to your participation? Is there anything that could be improved or done to reduce your burden? Do you want to leave some other comments on something that you think is worthwhile or important?*

### Plans to promote participant retention and complete follow-up {18b}

The study was designed to minimize as much as possible the burden for the participants. The entire procedure will be completed in a period of two hours during which the investigator will always be present in the room to assist the participant with all potential problems or difficulties experienced. In case of discontinuation of participation for any reason, the participant will be asked to complete the PQE (see above) to further assess the reasons for discontinuation if not directly expressed by the participant or evident to the investigator. A follow-up visit will be offered to the participant in case of discontinuation due to AE.

### Data management {19}

eCRF entries will be considered source data since the CRF is the site of the original recording (e.g., there is no other written or electronic record of data). In this study, the Qualtrics Survey module will be the eCRF and will be used as the source document for the anamnestic information collected at the screening and for all the assessments and evaluations.

All study data will be entered on the online Qualtrics Survey. Data will be protected in accordance with the “Qualtrics Security statement” (SOC 2 Type II Certification, ISO 27001, 27017, and 27018 Certifications, FedRAMP Authorization, HITRUST), and access available only to the research group. The data will be stored in the online Qualtrics Account owned by to the Principal Investigator (PI) and protected by a unique password.

The participants will be identified by a unique sequential study-specific participant ID number and a unique randomization code. These two numbers will be recorded in the participant module in the IMotions software [32] and on the first page of the online Qualtrics Survey [31]. The name and any other identifying detail will NOT be included in any study data electronic file and no key between participant ID number, name, or other information that could identify the participant will be used. The data recorded in the IMotions software will be stored off-line in a dedicated computer, protected by a password that only the PI has access to.

All the other documents (informed consent, envelope with participant number and randomization code, MINI, and SCID 5 PD) will be stored in a locked cupboard at the lab accessible only to the PI. On all study-specific documents, other than the signed consent, the participant will be referred to by the study participant number/code, not by name.

### Confidentiality {27}

The trial staff will ensure that the participants’ anonymity is maintained. The participants will be identified only by the participant’s ID number on the eCRF and any electronic database. All documents will be stored securely and only accessible by trial staff and authorized personnel. The study will comply with the Data Protection Act 1998 which requires data to be anonymized as soon as it is practical to do so.

### Plans for collection, laboratory evaluation, and storage of biological specimens for genetic or molecular analysis in this trial/future use {33}

N/a. No biological specimens will be collected in this study.

## Statistical methods

### Statistical methods for primary and secondary outcomes {20a}

This study constitutes a randomized trial employing repeated measures of a continuous outcome. To address the repeated measures within individuals as delineated by the inclusion criteria (specifically Swedish-speaking females aged 18-50 years diagnosed with BPD), and considering the attendant challenges of collinearity and intra-individual variability, mixed models with individuals as random effects will be employed across various stages of the study (baseline, pre-induction, post-induction, and recovery), as recommended by prior literature [76]. Covariates collected at baseline will be considered, with treatment group, treatment state, and time entered as fixed effects to estimate the effects stemming from the sham/intervention at each time point. The mixed model is versatile, accommodating both continuous and dichotomous outcomes, and will be utilized as deemed appropriate. Given the multitude of potential variables and outcome measures in this study, the necessity for adjustment for multiple tests may arise. To address the issue of multiple testing, we will consider applying the Bonferroni correction, which involves dividing the desired alpha level (typically 0.05) by the number of tests conducted. Alternatively, we may employ false discovery rate (FDR) correction methods to account for the increased risk of false positives when conducting multiple comparisons. The specific method chosen will depend on the final number and nature of outcome measures and variables included in the analysis and will be determined following the completion of the protocol.

### Interim analyses {21b}

No interim analysis is planned.

### Methods for additional analyses (e.g., subgroup analyses) {20b}

The sample is small, so no subgroup analyses are planned a priori.

### Methods in analysis to handle protocol non-adherence and any statistical methods to handle missing data {20c}

In case of missing response to the PANAS at any post-induction stage, a mean value of other post-induction stage measurements will be used. Since the study is a single-session study under clinician oversight, we do not expect any missing variables.

### Plans to give access to the full protocol, participant level-data and statistical code {31c}

Direct access will be granted to authorized representatives from the sponsor, host institution, and the regulatory authorities to permit trial-related monitoring, audits, and inspections. In accordance with our study protocol, access to study data by independent researchers will be considered following the completion of the trial and publication of primary results. Requests for data access will be subject to review by the Principal Investigator and study team to ensure compliance with ethical and regulatory requirements, protection of participant confidentiality, and preservation of intellectual property rights. Access to data will be granted upon approval of a data-sharing agreement outlining the terms and conditions of data use, including appropriate acknowledgments and restrictions. We are committed to promoting transparency and scientific collaboration while safeguarding the interests of study participants and stakeholders.

## Oversight and monitoring

### Composition of the coordinating center and trial steering committee {5d}

This is an investigator-initiated study. The PI and research team will collectively manage all aspects of the research process, including protocol development, participant recruitment, data collection, analysis, and reporting. Although there isn’t a designated management team or external steering committee, internal mechanisms have been established to ensure rigorous oversight and adherence to ethical, regulatory, and institutional guidelines.

### Composition of the data monitoring committee, its role and reporting structure {21a}

The study will be monitored by a local extern monitor independent from PI. The results of monitoring will be reported to the sponsor.

### Adverse event reporting and harms {22}

All AE’s occurring during the study observed by the investigator or reported by the participant, whether or not attributed to the device under investigation will be recorded on the eCRF as specified in the protocol. All Adverse device effects (ADE) will be recorded in the eCRF.

The following information will be recorded: description, date of onset and end date, severity, assessment of relatedness to device, other suspect drug or device, and action taken. Follow-up information should be provided as necessary.

The relationship of AEs to the device will be assessed by a medically qualified investigator or the sponsor/manufacturer and will be followed up until resolution or the event is considered stable.

All ADEs that result in a participant’s withdrawal from the study or are present at the end of the study, will be followed up until a satisfactory resolution occurs.

The Sahlgrenska University Hospital and the Department of Psychiatry for Affective Disorders will undertake an initial review of the information and ensure it is reported to the manufacturer. Events will be followed up until resolution, any appropriate further information will be sent by the research team in a timely manner.

Reporting to Läkemedelsverket will be done in liaison with the Chief Investigator and the Manufacturer.

In addition to the above reporting the Chief Investigator will submit once a year, throughout the trial, or on request a progress/safety report to Sahlgrenska University Hospital and Västra Götaland Region.

### Frequency and plans for auditing trial conduct {23}

In accordance with the nature of this investigator-initiated study, the roles and responsibilities of the coordinating center and trial steering committee are fulfilled by the PI and the research team. The PI assumes leadership in all aspects of the study, overseeing protocol development, participant recruitment, data collection, analysis, and reporting. Internal mechanisms have been established to ensure thorough oversight and adherence to ethical, regulatory, and institutional guidelines. Regular team meetings and protocol reviews facilitate continuous monitoring of study progress and prompt action to address any issues or challenges encountered during the research process.

### Plans for communicating important protocol amendments to relevant parties (e.g., trial participants, ethical committees) {25}

The protocol, informed consent form, participant information sheet, and any proposed advertising material have been submitted to the appropriate Research Ethics Committee (Etikprövningsmyndighet), regulatory authorities (Läkemedelsverket), and host institution (Sahlgrenska University Hospital) for written approval. The Investigator will submit and, where necessary, obtain approval from the above parties for all substantial amendments to the original approved documents.

### Dissemination plans {31a}

All results will be published in peer-reviewed scientific journals and all contributing authors will be included in accordance with the Vancouver recommendations.

## Discussion

We have described our protocol for a randomized controlled trial to evaluate the efficacy of one taVNS session to acutely decrease emotional vulnerability and improve emotional regulation in patients with BPD.

To the best of our knowledge, this study represents the initial exploration into the acute effects of taVNS on the three facets of emotional vulnerability outlined in Linehan’s biosocial model [5] among individuals diagnosed with BPD. Furthermore, this investigation marks the first instance of evaluating taVNS in this population. We believe that our study will help to better understand the involvement of vagal tone in emotional vulnerability and regulation in BPD patients and possibly lead to the development of new treatment strategies.

TaVNS offers a non-invasive, portable, and user-friendly option, allowing for convenient “as needed” use. Notably, emotion dysregulation, stemming from emotional vulnerability, is a central feature of many serious BPD-related challenges, such as self-injury and impulsive behaviors. Addressing emotional vulnerability is a key aspect of Dialectical Behavioral Therapy (DBT) [5], one of the few therapies showing efficacy for the treatment of BPD [77]. Current approaches often involve off-label use of medications with significant side effects, as there are limited interventions available [78]. Given its safety profile and minimal side effects, taVNS could serve as a valuable adjunct to psychotherapy or medication, enhancing treatment options for individuals with BPD. If taVNS will be shown to be efficacious to acutely decrease emotional vulnerability and improve emotion regulation, future research should explore the potential of using taVNS alongside standard BPD treatments in real settings. Potential future studies could investigate the impact of an as-needed use of taVNS in adjunct to psychotherapy, on the frequency or severity of dysfunctional self-regulating behaviors like, for example, self-harm or drug abuse, in comparison with psychotherapy alone. Additionally, given that the present study will not give information on the long-lasting effects of taVNS, or on the effects of a prolonged taVNS use, on emotional vulnerability and regulation, it would be worthy to investigate the efficacy of a daily taVNS treatment, on these two dimensions and the related BPD symptoms. Finally, in case of acute efficacy of taVNS on emotional vulnerability and/or emotional regulation, there could be a potential for its application in the treatment of other psychiatric disorders characterized by emotional dysregulation and autonomic imbalance, as PTSD or complex-PTSD.

## Trial status

tVNS-BPD-001, 10/OCT/2022 v.1.0. The recruitment began the 24/MAR/2023 and is expected to be completed the 31/SEP/2025

## Data Availability

Direct access will be granted to authorized representatives from the sponsor, host institution, and the regulatory authorities to permit trial-related monitoring, audits, and inspections.

## Abbreviations

ACC: Anterior cingulate cortex
ADE: Adverse device effect
AE: Adverse event
BPD: Borderline personality disorder
BSL-23: Borderline Symptom List 23
BSL-SUPP: Borderline symptom list - behavior supplement
CAN: Central autonomic network
DERS: Difficulties in Emotion Regulation Scale
dlPFC: Dorsolateral prefrontal cortex
ECG: Electrocardiogram
eCRF: Electronic case report form
EMA: European Medicines Agency
FDA: Food and Drug Administration
GABA: Gamma-aminobutyric acid
GSR: Galvanic skin response
HRV: Heart rate variability
iVNS: Invasive vagus nerve stimulation
MDD: Major depressive disorder
MINI: The Mini International Neuropsychiatric Interview
mPFC: Medial prefrontal cortex
NIM: Neurovisceral integration model
NTS: Nucleus tractus solitarii
PANAS: Positive and Negative Affect Schedule
paVNS: Percutaneous auricular vagus nerve stimulation
PEME: Perceived effectiveness in managing emotions
PI: Principal Investigator
PQE: Participation Quality Evaluation
PVT: Polyvagal theory
rACC: Rostral anterior cingulate cortex
SCID-5-PD: Structured Clinical Interview for DSM-5 Personality Disorders
sgACC: Subgenual anterior cingulate cortex
SIL: Subject Information Leaflet (see PIL)
taVNS: Transcutaneous auricular vagus nerve stimulation
tcVNS: Transcutaneous cervical vagus nerve stimulation
tVNS: Transcutaneous vagus nerve stimulation
VNS: Vagus nerve stimulation
